# Deformation Detection Method of Mine Tunnel Based on Mobile Detection System

**DOI:** 10.3390/s20185400

**Published:** 2020-09-21

**Authors:** Jian Sun, Haili Sun, Ruofei Zhong, Yulong Han

**Affiliations:** 1 Beijing Advanced Innovation Center for Imaging Theory and Technology, Key Laboratory of 3D Information Acquisition and Application, MOE, College of Resource Environment and Tourism, Academy for Multidisciplinary Studies, Capital Normal University, Beijing 100048, China; 2180902101@cnu.edu.cn (J.S.); zrf@cnu.edu.cn (R.Z.); 2190902205@cnu.edu.cn (Y.H.); 2 College of Resource Environment and Tourism, Capital Normal University, Beijing 100048, China

**Keywords:** mining method, tunnel, mobile laser scanning, clearance detection, multi-sensor

## Abstract

Subway structure safety detection is an important method to ensure the safe operation of trains. Efficient, high-precision, and automatic tunnel clearance detection is the key to ensure safe operations. This study introduces a mobile tunnel scanning system that integrates a scanner, an inertial measurement unit (IMU), and a rail car. Global Navigation Satellite System (GNSS) time and system hardware calibration are used to synchronize time and space information of the system; the attitude and speed are corrected using the control points from the tunnel to improve the accuracy of absolute positioning. The section coordinate system is converted using the control points and system calibration parameters to complete the tunnel clearance inspection, and the distance between the nearest point of the section and the clear height of the vault is given. Taking Fengxi Road’s Bashan tunnel section of Chongqing Metro Line 5 as an example, the overall system accuracy was tested. The accuracy of chord line measurements was within 1 mm, the internal coincidence accuracy of repeated measurements of the vault clear height was 1.1 mm, the internal coincidence accuracy of repeated measurements of the closest gauge point was 4.8 mm, and the system calibration accuracy was approximately 2 mm. Compared with the existing scheme, the system combines absolute measurement and relative measurement mode to judge the structural safety of tunnel section from multiple angles, high precision, and high efficiency.

## 1. Introduction

China has undergone rapid development in urban rail transit; in 1965, the first subway was built in Beijing and, by the end of 2019, the nation’s urban rail transit lines reached 6736.2 km, including 5180.6 km of metro lines (76.9%). In 2019, the length of newly added operation lines was 974.8 km, with a total daily passenger traffic volume of 66.371 million persons; the average daily passenger traffic volume of Beijing and Shanghai numbered more than 10 million persons. However, according to incomplete statistics, 1416 delay events of 5 min or more occurred in 2019 [1]. Because of the influences of geology, stress, and groundwater changes, internal deformation will inevitably occur in tunnels at different degrees. Therefore, to ensure normal subway operations and ensure the safety of people’s lives and property, it is especially important to monitor the deformation of the lining ring in a tunnel. Tunnel clearance detection is a part of tunnel deformation monitoring. To ensure that the pipeline and other accessories in a tunnel do not intrude the vehicle gauge, the normal operation of vehicles can be judged for being disturbed or damaged by the positional relationship between the gauge and objects, such as pipeline equipment in the tunnel.

Moreover, owing to the continuous shortening of the operation time window during operation periods, efficient and accurate tunnel clearance detection methods have always been an important subject of metro operation safety [2,3,4].

Over many years, several ideas have been proposed for tunnel deformation detection. To detect tunnel lining cracks and other state information, the contact detection method was adopted. In this method, an extensible mechanical arm is installed on the detection vehicle, and the tunnel lining state is measured by mechanical vibration. If there is a boundary invasion, the equipment will sound an alarm. Because of the limitations of the equipment itself, this method is prone to equipment damage or providing an insufficient measurement range [5]. In the noncontact detection method, the total station, measuring robot [6,7], laser profiler, three-dimensional laser scanner, and other equipment are often used. In the application of a total station in tunnel clearance detection, through observing the fixed positions at the top and bottom of tunnel section, the transverse, longitudinal, and settlement deformations of the tunnel section are calculated to predict the rock mass change. Because of the low density of collected cross-section points, it is difficult to detect an entire section, and the operation efficiency is low [8,9]. The application of a laser profiler in the quality inspection of tunnel lining can aid in completely obtaining information for an entire section, but the laser profiler must always be perpendicular to the track center line, which is difficult to achieve in practice [10]. The station 3D laser scanner can obtain high-precision, point cloud information in a tunnel with a high density, which can accurately reflect the real state of the tunnel. However, in data processing, the algorithm is relatively complex, requiring multi-station splicing and cross-section extraction. In the actual operation process, station-to-station observations are also required, which leads to a low operation efficiency and heavy postprocessing workload [11,12,13,14,15,16,17,18,19]. From the aspect of multi-sensor fusion, Liu et al. [20] designed a tunnel monitoring car integrating two laser ranging sensors, four displacement sensors, an odometer, and a point laser, and used Peripheral Component Interconnect (PCI) and computer real-time communication to display the measured information of a tunnel section. Zhou et al. [21] integrated a laser scanner and a high-precision navigation and positioning system on a tunnel car. Through the track extraction of a three-dimensional point cloud, the track center was fitted and the tunnel section clearance was calculated. Du et al. [22] placed a laser scanner, odometer, and displacement sensor on a track car to detect the deformation of a tunnel section. Boavida et al. and Puente et al. [23,24] developed a set of systems for clearance detection of highway tunnels. A lidar scanner, GNSS antenna, inertial measurement unit, and odometer were assembled on a vehicle for a clearance detection of a highway tunnel. However, owing to the vibration of the vehicle body, the accuracy of the clearance detection was low. The Amberg company of Switzerland developed GRP5000 track detection car [25] that included a PROFILER5002/5003 3D laser scanner, displacement sensor, inclinometer, and odometer. Their system demonstrated an acceptable performance in gauge detection, completion acceptance, holographic image acquisition, and so on. Developed by Leica company, the SiTrack:One track movement detection system [26] integrates a P40 scanner, noncontact laser odometer, high-precision inertial navigation measurement unit, and GNSS antenna; this system is suitable for existing outdoor railways as well as metro tunnels without a GNSS signal. Through using postprocessing software, a relative point cloud accuracy of 3–4 mm can be obtained and the track can be extracted automatically, mileage can be calculated, and collision detection and detection reports can be generated automatically. Hao et al. [27] proposed a mobile laser scanning system for shield tunnels, extracted tunnel appendages through wavelet filtering, fitted ellipses, calculated tunnel convergence diameter, and generated a gray image to detect tunnel circumferential cracks. Du et al. [28,29,30,31] analyzed the cross-section deformation of a shield tunnel from the aspects of tunnel section extraction, convergence diameter calculation, and generated a gray image of a tunnel point cloud using a mobile laser scanning system that integrated scanner and odometer. Researchers have obtained tunnel information via laser point clouds and have attained certain achievements in the development of digital imaging technology [32,33,34]. However, owing to the complexity of digital image acquisition, the accuracy of tunnel structure data cannot be guaranteed and the lack of light in tunnels will also have a certain impact on data acquisition.

In the above research on tunnel clearance detection methods, owing to the low detection efficiency of total stations and other equipment, the postprocessing workflows of station scanner data are excessively complex. However, the working mode, software, and hardware cost of the tunnel detection system developed by Amberg company and Leica company, as well as its applicability to the Chinese market, must be improved. There is a lack of relevant research on tunnel deformation detection using the mining method. Therefore, an efficient, high-precision, cost-effective tunnel detection system is increasingly necessary. This study introduces a tunnel detection system that focuses on the detection of tunnel section deformation using the mining method. The system integrates a laser scanner, inertial measurement unit, and GNSS antenna, and is mounted on a rail car that can travel at a uniform speed. Outdoors, the GNSS time received by GNSS antenna is synchronized with the time system of the scanner and IMU through a Pulse Per Second (PPS) signal. Combined with setting control points, the system attitude angle and car speed are calibrated. Through the control points and system calibration parameters to calculate the track center, the point cloud data and inertial navigation data are combined to calculate the tunnel clearance to determine whether there is deformation inside the tunnel.

## 2. Materials and Methods—Design and Implementation of Tunnel Mobile Scanning System

This study introduces a type of tunnel moving scanning system that can be applied to shield tunnel and mine tunnel clearance convergence detections.

### 2.1. Hardware Integration and System Synchronization

The tunnel mobile scanning system is mainly composed of Leica scanner P16, the NovAtel inertial navigation system, a rail car, notebook computer, and other parts. The Leica P16 scanner has a range accuracy of 1.2 mm + 10 parts per million (ppm), an angle accuracy of 8 “, and a field-of-view angle of 360° in the horizontal direction and 290° in the vertical direction. The scanning mode adopts cross-section scanning. The SPAN-FSAS inertial navigation system mainly includes a GNSS antenna, a NovAtel receiver card, and an inertial measurement unit and battery. The inertial measurement unit adopts a closed-loop fiber optic gyroscope with an acquisition frequency of 200 Hz. The rail car contains an electric motor and odometer that can run constantly on the track at five different speeds: 0.05 m/s, 0.20 m/s, 0.5 m/s, 1.0 m/s and 1.5 m/s.

The system synchronization mainly includes time synchronization and space synchronization. To ensure that the scanner and IMU in the system are consistent in the time system, the scanner, IMU, and rail car are consistent in the space coordinate system. Therefore, it is necessary to unify the time and space coordinate system of each sensor in the system.

**Figure 1:**
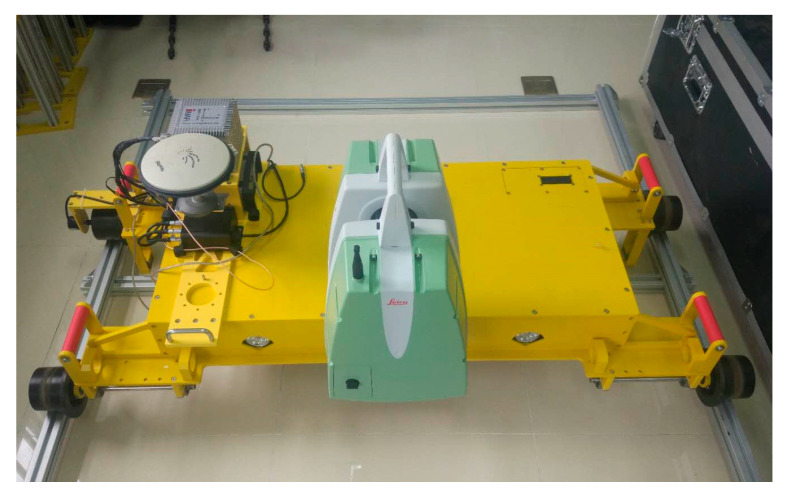
System integration.

In terms of time synchronization, the system unifies the time of scanner and IMU through the satellite signal received by GNSS. As shown in Figure 1, with the propak-v3 receiver as the center, the GNSS antenna transmits the received satellite ephemeris data and original observation data to the receiver in real time through the GNSS port, and SPAN-FSAS transmits the original observation data of inertial navigation system to the receiver in real time through the Component Object Model three (COM3); concurrently, the receiver transmits time synchronization information to the scanner and IMU through the COM2 and COM3 ports; the input/output (I/O) port is responsible for sending the PPS signal to IMU and the scanner to determine the accurate time, and then the receiver transmits the time stamp of this accurate time to IMU and scanner through COM2 and COM3 at a frequency of 1 Hz. Finally, the receiver transmits the satellite original observation data and IMU original observation data to the computer through the COM1 port.

In space synchronization, this study used the method of system calibration to unify the spatial coordinate system. By calibrating the scanner, the scanner coordinate system and the vehicle body coordinate system are unified; by calibrating the inertial navigation system, the vehicle body coordinate system is unified with both the rail surface coordinate system and the IMU coordinate system. The system uses a constant-speed rail car, calculates the mileage through the car speed, and can directly generate three-dimensional relative point cloud without a scanner calibration and inertial navigation calibration.

### 2.2. System Calibration

In the actual measurement, to reduce the error caused by multi-sensor fusion, scanner calibration, inertial navigation calibration, and rail car speed calibration can be performed beforehand.

#### 2.2.1. Scanner Calibration Principle

The scanner calibration is used to calculate the conversion parameters between the scanner coordinate system and the vehicle body coordinate system with the aid of the total station and reflector. First, four reflectors are pasted on the same section of the tunnel wall, and three reflectors are pasted on the rail car. The coordinates of the centers of the seven reflectors are measured by the total station, and the scanner begins to scan the tunnel.

In the calculation of calibration parameters, we defined the vehicle coordinate system (VC), scanner coordinate system (LC), and total station coordinate system (TC). First, the conversion parameters between the TC and the LC were calculated through the four reflector center points on the tunnel wall [35]. Second, the conversion parameters between TC and VC were calculated through three common points on the vehicle body. Finally, the conversion parameters between the LC and VC were obtained through two translations and rotations. Subsequently, the point cloud in LC was converted to VC.

#### 2.2.2. Static Calibration of IMU Attitude Angle

**Figure 2:**
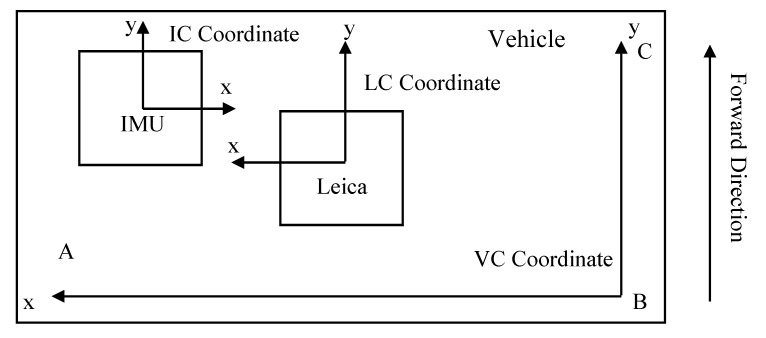
Schematic of each sensor coordinate system.

In this subsection, we define the inertial navigation coordinate system as IC. As shown in Figure 2, owing to the influence of the actual installation error, the IC and VC cannot completely coincide, so the attitude angle of IMU must be calibrated statically.

In this study, the IMU attitude angle static calibration used the total station to measure the coordinates of tunnel engineering coordinate systems of A, B, C on the rail car body. According to Equations (1)–(3), the attitude angle of the car at that time can be obtained. Then, compared with the attitude angle observed by IMU, the difference of the three-axis attitude angle between the IC coordinate system and the VC coordinate can be obtained. The average value is taken as the final result of IMU attitude angle static calibration, so the IMU coordinate system can be placed into the VC.

(1) $$Yaw=arctan\left(\frac{y_{c}-y_{b}}{x_{c}-x_{b}}\right)$$

(2) $$Pitch=arcsin\left(\frac{z_{c}-z_{b}}{\sqrt{{\left(x_{c}-x_{b}\right)}^{2}+{\left(y_{c}-y_{b}\right)}^{2}+{\left(z_{c}-z_{b}\right)}^{2}}}\right)$$

(3) $$Roll=arcsin\left(\frac{z_{b}-z_{a}}{\sqrt{{\left(x_{b}-x_{a}\right)}^{2}+{\left(y_{b}-y_{a}\right)}^{2}+{\left(z_{b}-z_{a}\right)}^{2}}}\right)$$

#### 2.2.3. Calibration of Rail Surface Inclination

In the actual tunnel environment, the plane of the rail car body cannot be completely parallel to the track surface, i.e., there is an angle between the rail surface coordinate system and the VC that causes a certain degree of system error for the later clearance detection. Therefore, the included angle should be calibrated before the actual operation.

**Figure 3:**
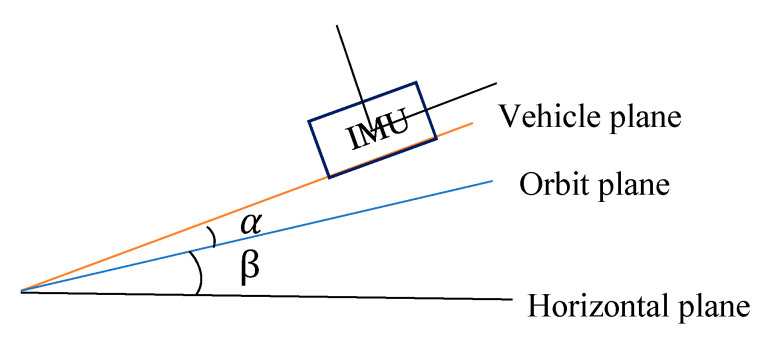
Schematic of direct survey.

**Figure 4:**
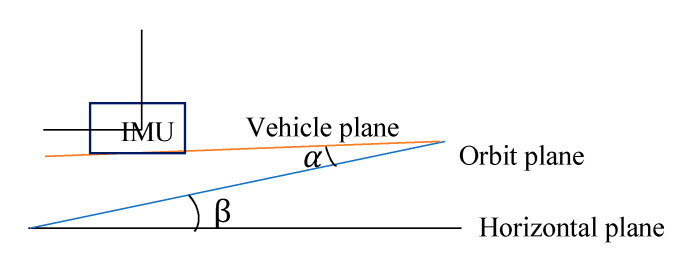
Schematic of reversed survey.

Back-and-forth measurements are used to calibrate the inclination of the rail surface. As shown in Figure 3 and Figure 4, α is the angle between the car body coordinate system and the rail surface coordinate system, and β is the actual inclination angle of the track. Before the actual tunnel operation, the IMU roll angle measurement is carried out every interval of a certain distance, and the car is rotated 180° to re-observe the roll angle at these five positions. Let the direct measured roll angle be $A_{1}=-\left(\alpha +\beta \right)$ and the reversed measured roll angle be $A_{2}=\left(\beta -\alpha \right)$; subsequently, the correct inclination angle is $\beta =(A_{2}-A_{1})/2$ and the included angle of coordinate system is $\alpha =\beta -A_{2}$. Therefore, we obtain the angle α between the car body coordinate system and the rail surface coordinate system to ensure that the point cloud under the car body coordinate system can be unified into the rail surface coordinate system.

#### 2.2.4. Speed Calibration

To reduce the impact of the accumulated error of mileage on the accuracy of point cloud fusion, it is necessary to calibrate speed. Although the rail car is driven by an electric motor at a constant speed, there is a gap between the nominal value and the real value because of the influence of nominal error and actual track conditions.

In this study, the speed calibration was divided into two parts: Field calibration and postprocessing. The field calibration was used to provide multiple calculations for the speed of the car by measuring the distance and the corresponding travel time. The average value of multiple observations is taken as the real speed value of the car to determine the speed of the rail car in the actual operation process. In the postprocessing part, the speed was corrected through the known control points in the tunnel. The specific content is described in detail in Section 4.1.

### 2.3. Generation Principle of 3D Relative Point Cloud

After the time synchronization between the scanner and IMU the 2D cross-section point cloud collected by the scanner, the attitude angle of IMU, and the mileage calculated by velocity and time can be fused together to generate a 3D relative point cloud with the same coordinate system. Next, we introduce the integrated navigation algorithm of the attitude angle and mileage in a 3D relative point cloud fusion.

In general, the scanner in the mobile scanner system adopts a section scanning mode, and each section point cloud is a relatively independent coordinate system. To unify the coordinate system of all section point clouds and restore the true line shape of the tunnel, we used the method of attitude angle and mileage to calculate the coordinate system transformation parameters of each section relative to the section at the starting position; this was done to convert the coordinates of the point cloud of each section to the coordinate system of the starting position section and, thus, generate the relative point cloud.

In this study, the tunnel mobile scanning system relied on GNSS time to unify the time system of inertial navigation and the scanner. To further unify the time tag of the point cloud section and IMU attitude angle, the interpolation method is usually used to calculate a continuous function according to the known discrete data and, thus, estimate the function value of other positions. The Lagrange interpolation method was used to calculate the corresponding attitude angle under the time label based on the time stamp of each section point cloud to ensure that we provided each section the attitude angle at the corresponding time.

After completing the attitude angle interpolation, we combined the scanner point cloud data, IMU attitude angle, and mileage value to generate a 3D point cloud with a relatively uniform coordinate system.

First, the time of the starting section was recorded as t1 and the time of the current section was recorded as t2. The difference of heading angle, pitch angle. and roll angle between t2 and t1 was calculated to ensure that each section had an attitude angle difference relative to the starting section.

**Figure 5:**
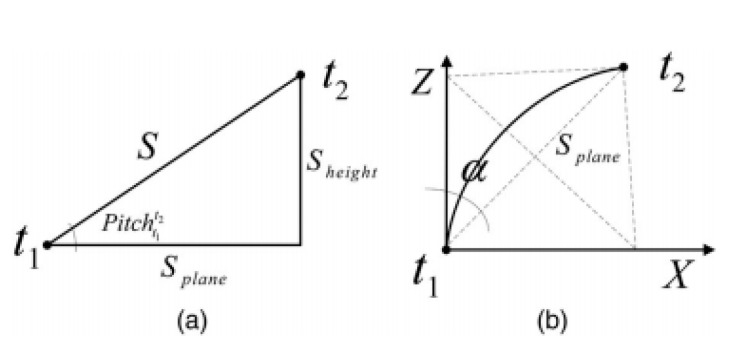
Calculation of translation parameters between two sections.

Subsequently, the translation parameters of coordinate transformation were calculated according to mileage difference, heading angle difference, and pitch angle difference as shown in Figure 5. The translation matrix $T_{t1}^{t2}$ of each section relative to the starting section can be calculated according to Equations (4)–(9).

(4) $$\alpha =Yaw_{t1}^{t2}/2$$

(5) $$S_{\mathrm{plane}}=S*\mathrm{Cos}\left({\mathrm{Pitch}}_{t1}^{t2}\right)$$

(6) $$Y_{t1}^{t2}=S*Sin\left(Pitch_{t1}^{t2}\right)$$

(7) $$X_{t1}^{t2}=S_{plane}*Sin\left(\alpha \right)$$

(8) $$Z_{t1}^{t2}=S_{plane}*Cos\left(\alpha \right)$$

(9) $$T_{t1}^{t2}=\left[\begin{array}{c} X_{t1}^{t2}\\ Y_{t1}^{t2}\\ Z_{t1}^{t2} \end{array}\right]$$

According to the three attitude angle differences of each section, the rotation matrix $R_{t1}^{t2}$ between the current section and the starting section can be obtained by Equation (10).

(10) $$R_{t1}^{t2}=\left[\begin{array}{ccc} Cos\left(R\right)\cos\left(Y\right) & Cos\left(R\right)Sin\left(Y\right) & Sin\left(R\right)\\ Sin\left(R\right)Cos\left(Y\right)Sin\left(P\right)-Sin\left(Y\right)Cos\left(P\right) & Sin\left(R\right)Sin\left(Y\right)Sin\left(P\right)+Cos\left(Y\right)Cos\left(P\right) & -Cos\left(R\right)Sin\left(P\right)\\ -Sin\left(R\right)Cos\left(Y\right)Cos\left(P\right)-Sin\left(Y\right)Sin\left(P\right) & -Sin\left(R\right)Sin\left(Y\right)Cos\left(P\right)+Cos\left(Y\right)Sin\left(P\right) & Cos\left(R\right)Cos\left(P\right) \end{array}\right]$$

$$\left(R={Roll}_{\mathrm{t1}}^{\mathrm{t2}},Y={Yaw}_{\mathrm{t1}}^{\mathrm{t2}},P={Pitch}_{\mathrm{t1}}^{\mathrm{t2}}\right)$$

Finally, according to Equation (11), each two-dimensional section was unified into the coordinate system of the starting section through translation and rotation, thus generating a 3D relative point cloud based on the starting section coordinate system.

(11) $$\left[\begin{array}{c} X_{t2}\\ Y_{t2}\\ Z_{t2} \end{array}\right]=R_{t1}^{t2}\left[\begin{array}{c} X_{t1}\\ Y_{t1}\\ Z_{t1} \end{array}\right]+T_{t1}^{t2}$$

## 3. Materials and Methods—Clearance Detection

Both in the mining method tunnel and the shield tunnel, the structural safety detection in the tunnel section has always been a considerably important problem during subway operations. This study evaluated the safety of the internal structure of the tunnel from three aspects: Chord line measurement, clearance detection, and arch crown clear height.

### 3.1. Chord Measurement

A line segment connecting any two points on a circle is usually called a chord. In the tunnel, because the tunnel lining is circular, we refer to the line segment formed by any two points in the tunnel section as the chord line. The chord line measurement method selects two points on both sides of the waist of the tunnel section to lay reflectors. Through the measurement and calculation of the tunnel mobile scanning system, the three-dimensional relative point cloud was obtained, the relative three-dimensional coordinates of the four reflectors were extracted, and each distance between the four points was calculated. A comparison of multi-stage, repeated detection data reflects whether the lining deformation occurred in the cross-sectional direction of the tunnel.

To evaluate accuracy, we used the total station to measure the three-dimensional coordinates of the four points in the tunnel and calculate the distance of the corresponding chord line. A comparison of the total station data was then used to determine the accuracy.

### 3.2. Gauge Detection

**Figure 6:**
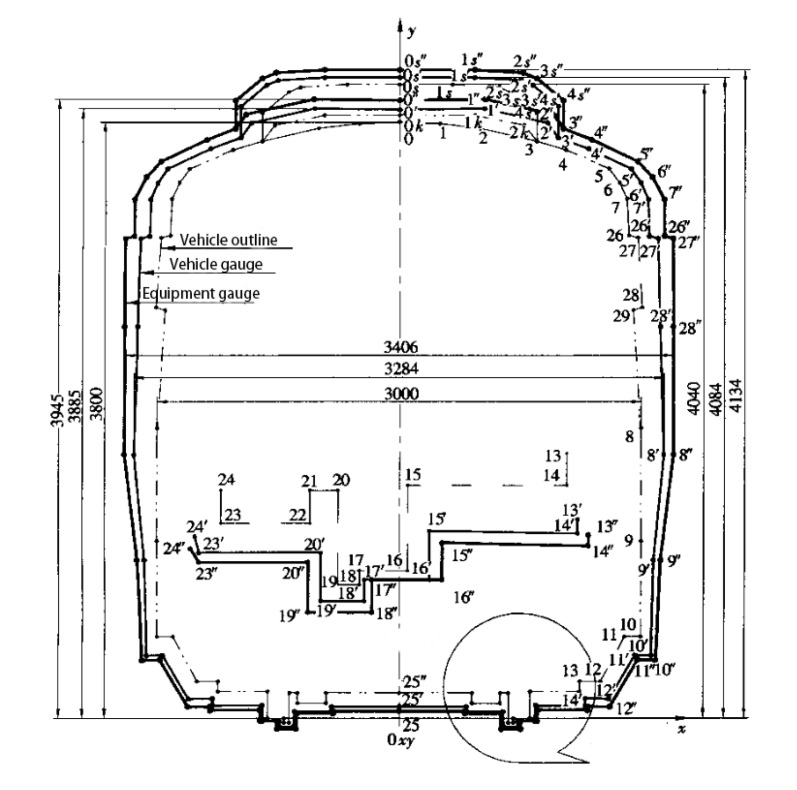
Metro vehicle gauge standard [36].

Clearance is a graph used to ensure the safe operation of subways, limit the size of vehicle sections, limit the installation size of equipment along the line, and determine the effective size of a building structure. According to different functional requirements, metro clearance is divided into vehicle clearance, equipment clearance, and construction clearance as shown in Figure 6.

The maximum dynamic envelope of the vehicle under normal operation is a straight-line section. “Normal operation” refers to the primary suspension and secondary suspension being within the normal elastic range, and the wearing of vulnerable parts is beyond the limit. The vehicle gauge of elevated or ground line is easily affected by the wind load, which must be considered in a calculation.

An equipment gauge is the dynamic envelope line when the primary suspension or secondary suspension fails during vehicle operation and is used to restrict the installation equipment from invasion.

The construction gauge is the minimum effective section after considering the installation size of equipment and pipeline based on the equipment gauge. Construction clearance does not include the measurement error, construction error, structural settlement, displacement deformation, and other factors.

In the operation phase of subway detection, mainly for vehicle clearance detection, detecting the presence of equipment or other objects in the vehicle contour line can ensure the safety of subway operations. Three types of vehicles (A, B1, B2) are defined in the metro design code. This paper considers type A vehicles as an example to explore the method of clearance detection.

#### 3.2.1. Calculation of Coordinate Transformation Parameters

To convert the section and point cloud from the VC to the rail surface coordinate system as well as unify the vehicle contour coordinates and the section point cloud coordinates, it is necessary to calculate the rotation and translation parameters.

According to the static calibration of inertial navigation attitude angle in Section 2.2.2, we can obtain the difference of three attitude angles between the IMU coordinate system and vehicle body coordinate system and correct the attitude angle of IMU. According to the content of the rail surface inclination calibration mentioned in Section 2.2.3, we can obtain the included angle α between the rail car and the rail surface; α is the rotation angle between the car body coordinate system and the rail surface coordinate system of the cross-section point cloud in the two-dimensional plane, and the two-dimensional rotation matrix is calculated according to equation $R=\left[\begin{array}{cc} cos\alpha & sin\alpha \\ -sin\alpha & cos\alpha \end{array}\right]$.

To obtain the translation transformation parameters between the car body coordinate system and the rail surface coordinate system, it was necessary to determine the relative position of the track center point in the section point cloud coordinate system. However, owing to the object occlusion on both sides of the rail car, the rail data could not be collected, so we used the control point correction method to determine the coordinates of the track center point.

First, we measured the vertical distance from the origin of the vehicle coordinate system to the lowest point of the wheel of the rail car. Because the height between the origin of the vehicle coordinate system and the contact surface between the wheel and the rail is fixed, the elevation coordinate Z0 of the track center point in the vehicle coordinate system can be determined. Then, the point cloud data of the same control point are collected through round-trip observations in the tunnel. By comparing the Y value of the control point with the Y value of the reversed survey control point, the forward measurement is assumed to be Y1 and the back measurement is Y2.

The y-axis coordinate value Y0 of the orbit center point can be obtained using equation Y0=(Y1+Y2)/2. We, thus, determined the coordinates of the track center point in the vehicle coordinate system (Y0, Z0). The translation matrix T was determined according to equation $T=\left[\begin{array}{c} -Y0\\ Z0 \end{array}\right]$.

According to equation $Point_{G}=R\cdot Point_{V}+T$, the section point cloud can be transformed from the vehicle coordinate system to the rail surface coordinate system, thus unifying the coordinate system of the section point cloud and the vehicle contour line.

#### 3.2.2. Gauge Detection Principle

According to the method mentioned in Section 3.2.1, we converted the section point cloud to the rail surface coordinate system; according to the metro design specifications [37], the coordinates of the vehicle contour line can be obtained, and the origin of the coordinate system should coincide with the center point of the track. Thus, the vehicle contour in the same two-dimensional coordinate system and the tunnel point cloud of the section are obtained. Next, we must judge the relationship between each point in the section point cloud and the vehicle contour.

**Figure 7:**
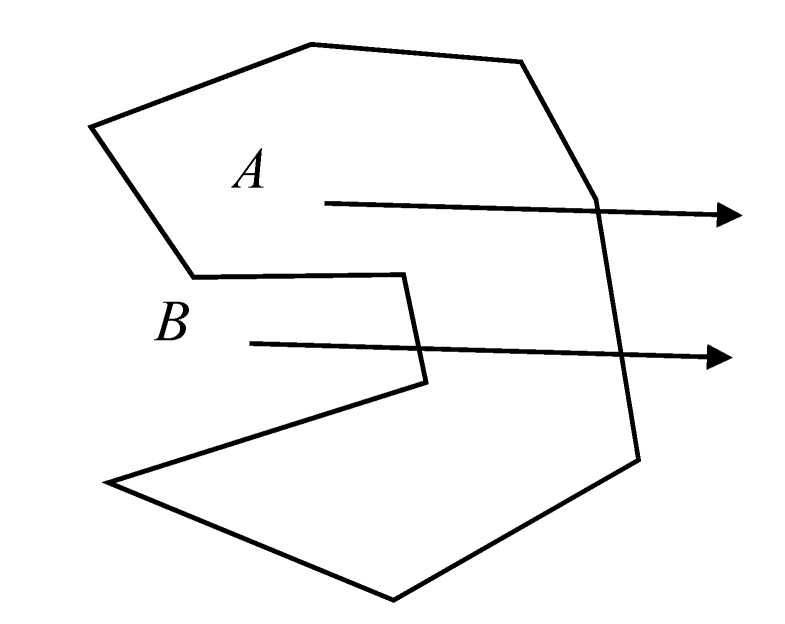
Horizontal ray method.

The limit detection method in the text mainly adopts the ray discrimination method [38]. To judge whether a point is in the polygon, a horizontal ray is emitted from this point as shown in Figure 7. If the number of intersection points between the ray and the polygon is an odd number, the point is within the polygon; if it is even, then the point is outside the polygon. Thus, the positional relationship between each point in the section and the vehicle contour line can be judged point by point and, thus, determine whether the wall of the tunnel and its appendages have invaded. If there is no invasion, the nearest point in the section to the vehicle contour line is calculated.

### 3.3. Calculation of Vault Clear Height

The clear height of a vault refers to the vertical height from the highest point in the tunnel to the track center point. By comparing the multi-stage repeated measurement data, the detection of the clear height of the arch crown can determine whether deformation of the top of the tunnel lining occurs.

In Section 3.2, we transformed the section point cloud to the rail surface coordinate system with the track center point as the origin. Then, according to the time label, the roll angle of each section at the corresponding time was added to the section coordinate system for correction, and the real attitude of the tunnel section was restored.

Assuming that the roll angle of the current section is α, the rotation matrix R is calculated by equation $R=\left[\begin{array}{cc} cos\alpha & sin\alpha \\ -sin\alpha & cos\alpha \end{array}\right]$ and the section point cloud under the rail surface coordinate system is rotated to the coordinate system in which the real roll angle attitude is restored, as shown in equation $Point_{new}=R\times Point_{old}$. The two-dimensional coordinate system takes the center point of the rail surface as the origin, and its Z axis is parallel to the Z axis of the tunnel engineering coordinate system, which can reflect the relative real elevation of each point in the section. Because the pitch angle is exceedingly small and does not affect the relative relationship of elevation, the pitch angle correction is not added.

In the point cloud of the tunnel section after restoring the real attitude, the point where the Z value is the maximum is the calculation point of the vault net height. However, owing to the influence of noise in the tunnel point cloud, it is necessary to denoise the cross-section point cloud. The fitting circle method and a given threshold are used to judge the noise points and unlined ring points in the top area. The circle fitting adopts the method of least squares and robust estimation to find the highest point of the tunnel lining ring [39,40], and the elevation value of this point is the clear height of arch crown. The deformation of tunnel top lining can be judged through a comparison of the multi-period repeated data.

## 4. Method Validation—Experimental Verification and Precision Analysis

**Figure 8:**
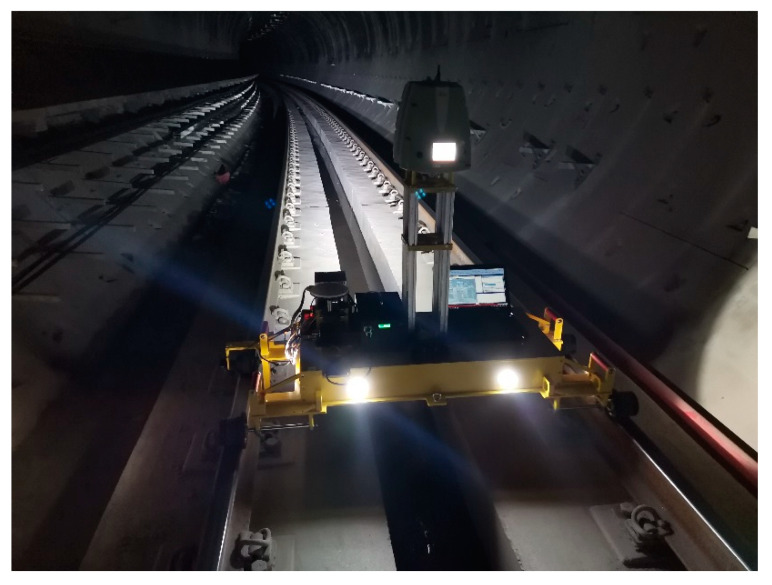
Field experiment environment.

This study takes the subway route from Bashan station to Fengxi Road Station of Chongqing Metro Line 5 as the test site, as shown in Figure 8. The tunnel part has been constructed by the mining method. The total length of the test area is approximately 60 m, is mainly a curve, and has known control points. The scanner calibration, inertial navigation calibration, and speed calibration were carried out in the field. The scanner data and inertial navigation data in the test area were collected by round-trip measurements, and reflectors were arranged on the site for attitude angle optimization, correction, and system calibration.

### 4.1. Attitude Angle Optimization and Velocity Correction

In the actual tunnel measurement, because the SPAN-FSAS integrated navigation system is in the satellite signal losing lock state for a lengthy period, its position accuracy, velocity accuracy, and attitude measurement accuracy have different degrees of decline. To improve the absolute positioning accuracy of the three-dimensional point cloud, the IMU attitude angle data should be optimized in the later data processing procedure.

#### 4.1.1. Attitude Angle Optimization

**Figure 9:**
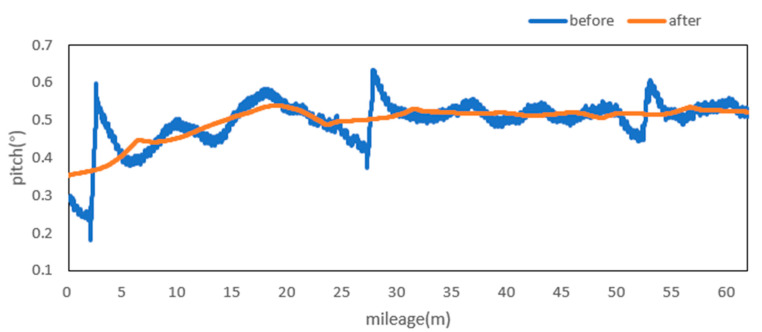
Pitch before and after moving the average filter.

**Figure 10:**
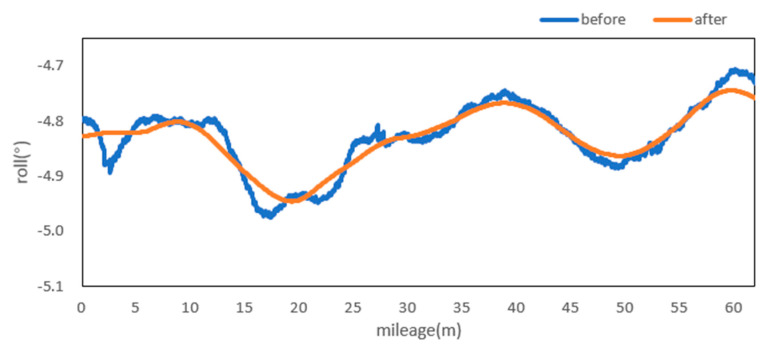
Roll before and after moving the average filter.

**Figure 11:**
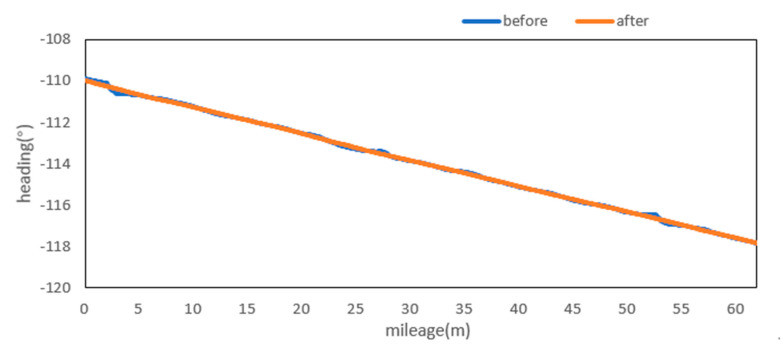
Heading before and after moving the average filter.

**Figure 12:**
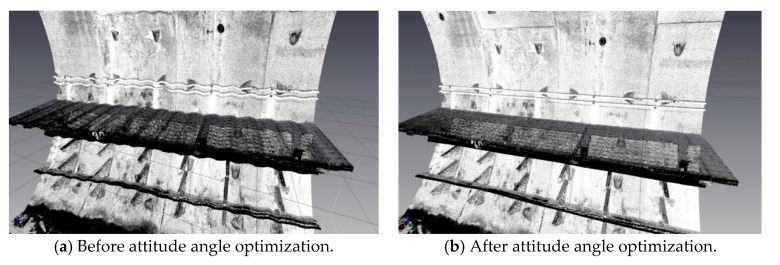
Tunnel point cloud data.

Attitude angle optimization is mainly divided into two parts: Filtering and correction. The moving average filter is used to filter the three attitude angles. Figure 9, Figure 10 and Figure 11 show the comparison curves of the pitch angle, roll angle, and heading angle before and after moving average filter, which shows that the attitude angle after filtering is more smooth and stable. Figure 12a,b shows the point cloud results obtained from the point cloud fusion of the two attitude angles before and after filtering at the same position. The more serious periodic point cloud change in Figure 12a can also be seen in Figure 12b; this change can eliminate the point cloud deformation caused by the random attitude angle error and the periodic system error caused by GNSS signal losing lock over a long period and restore the real structural information in the tunnel.

#### 4.1.2. Attitude Angle and Velocity Correction

In the process of tunnel operation, the attitude angle collected by IMU has a certain system error caused by the GNSS signal losing its lock state in the integrated navigation system, and an accumulated error in mileage will also be caused by the track car in the lengthy process of uniform driving. The reflector correction method is used to correct the attitude angle of integrated navigation and velocity by measuring the reflector coordinates on the starting and ending sections via the total station.

First, two sections were selected from the starting point and the end point of the test section in the tunnel. Four reflectors were arranged at the same position in each section, and the reflectors were evenly distributed on both sides of the tunnel wall. Second, the three-dimensional coordinates of eight reflectors in the two sections under the tunnel engineering coordinate system were collected using the total station. Then the mobile laser scanning system was started to collect the data of the test section.

The plane distance and coordinate azimuth between the corresponding points in the two sections were calculated. By comparing the total station data with the measured data of the scanner, four approximately equal angle differences and length differences were obtained. The average coordinate azimuth angle difference was 0.45° and the average distance difference was 21 cm. According to the average distance difference and the time difference between the starting and ending sections, a more accurate speed value of the rail car was calculated, and the heading angle was corrected according to the average angle difference and empirical estimation.

**Figure 13:**
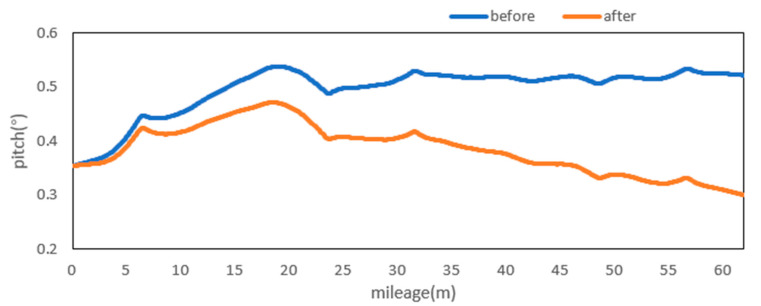
Pitch before and after optimization and correction.

**Figure 14:**
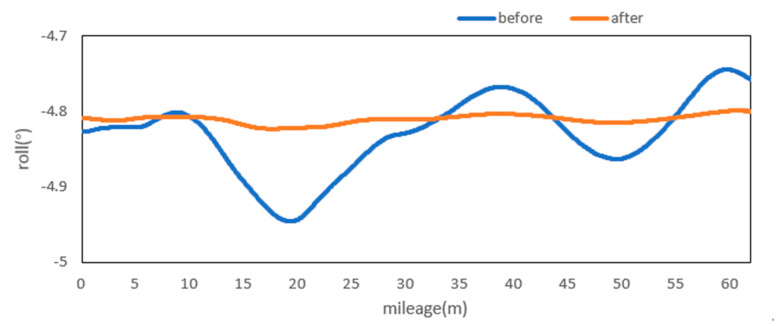
Roll before and after optimization and correction.

**Figure 15:**
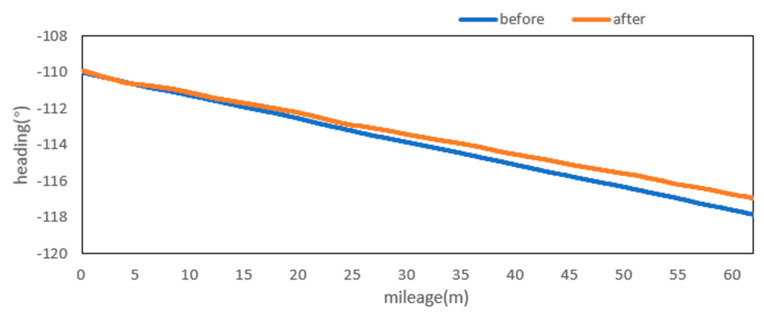
Heading before and after optimization and correction.

From the fusion formula of the relative point cloud, the influence of the pitch angle on plane line shape is relatively small. Figure 13 also shows that the pitch angle is approximately 0.4° to 0.5°, so the influence of the pitch angle on the plane line shape can be ignored in this experiment. The test section is located at the corner of the tunnel. The horizontal alignment is mainly a curve and the super elevation is relatively stable. Figure 14 shows that the roll angle fluctuates around −4.8°. To reduce the impact of periodic fluctuation, the fluctuation range of all roll angles in the test section was reduced 10 times, and the orange curve, as shown in Figure 14, was obtained. As shown in Figure 15, the heading angle deviation was corrected according to the average coordinate azimuth angle difference calculated above. The average angle difference of twice was evenly distributed to each heading angle in the test section, and the orange curve was obtained, as shown in Figure 15. The pitch angle had negligible influence on the plane line type, but its influence on the elevation was substantive. Therefore, the elevation angle was corrected according to the elevation difference between the starting and ending section reflectors. As shown in Figure 13, the orange curve is the corrected pitch angle.

At this point of the experiment, the three attitude angles and velocity corrections were computed. The three-dimensional relative point cloud was fused again with the corrected attitude angle and velocity and compared with the total station data; the absolute plane point position error of the reflector was reduced to less than 5 cm, and the elevation error to less than 2 cm. The smoothness and absolute coordinate accuracy of point cloud fusion show that the optimization and correction of attitude angle played a significant role.

### 4.2. Point Cloud Accuracy Analysis

This study analyzed and verified the reliability of the mobile laser scanning system in clearance detection from four aspects: System integration accuracy, chord line accuracy, arch crown clear height internal coincidence accuracy, and clearance detection internal coincidence accuracy.

#### 4.2.1. System Integration Accuracy

Table 1 shows the scanner calibration parameters and their accuracy. The scanner calibration calculates the conversion parameters between the scanner and the vehicle coordinate system by taking the coordinates of the total station as the transit coordinate system. The accuracy reflects the installation error of the scanner. The table shows that the mean square error of the conversion parameters was within 2 mm.

Table 2 shows the results of attitude angle calibration parameters. In the table, the difference between the inertial navigation attitude angle and the vehicle body attitude angle was calculated by comparing the total station data with the IMU data, so the IMU coordinate system was integrated into the vehicle coordinate system.

Table 3 shows the calibration parameter results of the rail surface inclination angle. The included angle between the track surface and car body plane was calculated by comparing the change of the roll angle in the round-trip measurement.

#### 4.2.2. Chord Accuracy

As shown in Table 4, chord lines 1–4 represent the chord length between reflectors 1 and 4 in the same section; for example, 1.1–4 represents the chord length between reflectors 1 and 4 in the first section. In comparing the data collected by total station and the data of point cloud fusion, we concluded that the accuracy of chord line was within 1 mm.

#### 4.2.3. Clear Height Accuracy of Arch Crown

The accuracy of the crown clear height is shown in Table 5. In the experimental range, 10 sections with different mileages were selected. In comparing the changes of the vault clear height at the same position in the round-trip measurement, we concluded that the internal coincidence accuracy of the vault clear height measurement was 1.1 mm.

#### 4.2.4. Gauge Detection Accuracy

The gauge detection precision is shown in Table 6. In the experimental region, 10 sections with different mileages were selected to calculate the distance of the closest point to the vehicle contour. By comparing the difference of the detection distance between the nearest point of round-trip measurement, we concluded that the internal coincidence accuracy of clearance detection was 4.8 mm.

## 5. Results

A detection method of tunnel deformation via the mining method, as based on a mobile scanning system, was proposed. The system integrates two kinds of sensors: Scanner and inertial navigation. The time system of scanner and IMU was unified by GNSS time. The spatial coordinate system was unified by calibrating the system, calibrating inertial navigation, calibrating the rails, and calibrating speed. The mileage of each section was calculated by constant-speed driving characteristics. It can be seen from Table 1 that the system integration accuracy was within 2 mm. Secondly, the optimization algorithm of 3D relative point cloud generation in mobile scanning system was introduced, and the attitude angle of IMU was filtered. It can be seen from Figure 9, Figure 10 and Figure 11 that the curve of pitch angle, roll angle, and heading angle with mileage before and after filtering tended to be smooth and stable. It can be seen from Figure 12a that the tunnel point cloud before attitude angle filtering had obvious spatial distortion and fluctuation, while, in Figure 12b, it can be seen that the tunnel point cloud after attitude angle filtering restored the real tunnel spatial structure. Finally, three kinds of clearance detection methods, including chord line measurement, vault clear height detection, and clearance closest point detection, were proposed to evaluate the structural safety of the tunnel from different perspectives, and the integration accuracy and clearance detection accuracy of the system were verified in Chongqing Metro Line 5 subway tunnel. Among them, eight groups of chord lengths were tested on site in the chord line measurement part, and the average values of these groups for the contrast chord length difference were taken as the evaluation precision. Table 4 shows that the accuracy was within 1 mm. Ten groups of vault clear heights at different positions were tested on site, and the average values of 10 groups of round-trip observation difference were taken as the final evaluation accuracy. Table 5 shows that the accuracy was approximately 1.1 mm. The distance of the nearest point in the section was tested at 10 different positions in the gauge detection part, and the average values of the differences between the 10 groups of round-trip observation were taken as the evaluation accuracy of the clearance detection. Table 6 shows that the accuracy was approximately 4.8 mm.

## 6. Discussion and Conclusions

According to the field test data, the value of the tunnel scanning system can be discussed from the following aspects. First of all, from the way of work, Leica’s SiTrack:One is integrated with scanners, inertial navigation, and odometer while Amberg’s IMS5000 not only integrates scanners, inertial navigation, and odometer, but also adds range finder and inclinometer, and the car is designed by hand. The tunnel scanning system mentioned in this paper uses the characteristics of electric car to drive at constant speed instead of odometer. The whole system only includes scanner and inertial navigation system, which can improve the efficiency and simplify the operation process, and the point cloud data generated by the electric vehicle is more uniform and stable. Secondly, from the perspective of measurement accuracy, the Leica SiTrack:One relative measurement accuracy was 3–5 mm, while the gauge measurement accuracy of Amberg IMS5000 was 5 mm and the section fitting accuracy was 3 mm. In this paper, the clearance detection accuracy was about 1 mm and the clearance detection accuracy was also kept within 5 mm. relative to SiTrack:One. When the absolute measurement accuracy was 8–10 mm, the absolute measurement accuracy in this paper needed to be improved. Finally, from the perspective of hardware cost, the cost of the tunnel detection system developed by Leica company and Amberg company is at the level of millions, which is difficult to promote and popularize in the current Chinese market. Compared with the existing tunnel detection system in China, such as only using scanner for tunnel deformation detection developed by Shanghai Exploration Institute, this system has a better improvement in measurement accuracy and operation content.

Generally speaking, the system can judge the structural safety problems in the tunnel from the horizontal, vertical, and nearest points of the tunnel, which can meet the needs of tunnel clearance detection with high efficiency and precision. In practical application, according to the repeated observation data of multiple periods, the location of excessive deformation in the tunnel was detected and the three-dimensional point cloud information of the deformation position was given, combining with the inertial navigation data. Due to the uniform speed of the rail car and the design features without odometer, the operation time and personnel can be reduced. Given that the detection times of skylights in the subway are increasingly shortening, our proposed method can improve the operation efficiency and save on manpower and material resources. At present, because the inertial navigation system and the rail car are powered separately, the overall equipment is excessively heavy and complicated, and it is difficult to travel up and down the tunnel in the process of field operations. The inertial navigation system can be packaged in the track car to reduce the unnecessary repetitive equipment. In addition, owing to the influence of attitude angle accuracy decline caused by the GNSS signal losing its lock over a long time and the cumulative error of mileage increasing with distance, the absolute coordinate accuracy of point cloud fusion in a tunnel still cannot meet the requirements of completion inspection. In the future, we will continue to study the correction of attitude angle and mileage by setting control point targets at equal distances to improve the absolute positioning accuracy of the entire point cloud.

## Figures and Tables

**Table 1:** Scanner calibration parameters.

Group	R			T	k	δ(mm)
1	1.000	−0.020	0.001	0.217	1.000	1.9
	0.020	1.000	−0.006	0.450		
	−0.001	0.006	1.000	0.904		

**Table 2:** Attitude angle calibration parameters.

Group	Roll (°)			Pitch (°)			Heading (°)		
	IMU	Station	△	IMU	Station	△	IMU	Station	△
1	4.261	4.445	−0.183	−0.141	−0.242	0.101	62.226	62.249	−0.023
2	4.257	4.477	−0.219	−0.140	−0.217	0.077	62.264	62.263	0.000
3	4.261	4.495	−0.234	−0.128	−0.179	0.051	62.265	62.221	0.045
4	4.264	4.476	−0.212	−0.119	−0.192	0.073	62.211	62.192	0.019
5	4.270	4.560	−0.289	−0.108	−0.179	0.071	62.154	62.123	0.031
Average			−0.228			0.075			0.015

**Table 3:** Calibration of rail inclination angle.

Group	Direct (°)	Reversed (°)	Inclination Angle (°)	Included Angle (°)
1	4.633	−4.587	4.610	−0.023
2	4.637	−4.600	4.619	−0.018
3	4.637	−4.613	4.625	−0.012
4	4.627	−4.624	4.625	−0.002
5	4.616	−4.628	4.622	0.006
Average			4.620	−0.010

**Table 4:** Chord accuracy.

Group	Total Station (m)	Point Cloud (m)	Difference (mm)
1.1–4	4.3881	4.3884	0.4
1.2–3	5.8006	5.8013	0.7
1.1–3	5.1650	5.1641	0.9
1.2–4	5.4029	5.4049	2.1
2.1–4	4.5209	4.5201	0.8
2.2–3	5.8868	5.8866	0.2
2.1–3	5.4968	5.4968	0.0
2.2–4	5.7253	5.7245	0.8
Average			0.7

**Table 5:** Clear height of arch crown.

Mileage (m)	Direct (m)	Reversed (m)	Difference (mm)
3.2	5.0588	5.0619	3.1
4.7	5.0627	5.0635	0.8
4.8	5.0630	5.0633	0.3
5.2	5.0648	5.0667	1.9
5.8	5.0680	5.0690	1.0
6.1	5.0705	5.0709	0.4
6.2	5.0734	5.0763	2.9
6.8	5.0749	5.0755	0.6
7.2	5.0769	5.0769	0.0
7.7	5.0801	5.0801	0.0
Average			1.1

**Table 6:** Gauge detection.

Mileage(m)	Direct (m)	Reversed (m)	Difference (mm)
3.2	0.3778	0.3707	7.1
4.7	0.3744	0.3693	5.1
4.8	0.3760	0.3694	6.6
5.2	0.3766	0.3708	5.8
5.8	0.3766	0.3719	4.7
6.1	0.3777	0.3733	4.4
6.2	0.3791	0.3734	5.7
6.8	0.3796	0.3766	3.0
7.2	0.3801	0.3769	3.2
7.7	0.3778	0.3753	2.5
Average			4.8

## References

[1] China Association of Metros Report on Statistics and Analysis of Urban Rail Transit in 2019. http://www.camet.org.cn/tjxx/5133.

[2] Shi H., Zhang Y. Study on management information system of railway line clearance detection based on noncontact measurement technique. Proceedings of the IEEE 2010 2nd International Conference on IIS.

[3] Seo D.J., Chool J. Development of cross section management system in tunnel using terrestrial laser scanning technique. Proceedings of the International Archives of the Photogrammetry, Remote Sensing and Spatial Information Sciences.

[4] Huang K.P., Wang T.-T., Huang T.-H., Jeng F.-S. (2010). Profile deformation of a circular tunnel induced by ambient stress changes. Tunn. Undergr. Space Technol..

[5] Haack A., Schreyer J., Jackel G. (1995). Report to ITA working group on maintenance and repair of underground structures: State-of-the-art of non-destructive testing methods for determining the state of a tunnel lining. Tunn. Undergr. Space Technol..

[6] Yao F., Shao G., Takaue R., Tamaki A. (2003). Automatic concrete tunnel inspection robot system. Adv. Robot..

[7] Yu S.N., Jang J.H., Han C.S. (2007). Auto inspction system using a mobile robot for detecting concrete cracks in a tunnel. Autom. Constr..

[8] Özbek A., Türkmen S., Gül M. (2003). The deformation evaluation of Kızlac, T3A tunnel (Osmaniye, Turkey). Eng. Geol..

[9] Kontogianni V., Tzortzis A., Stiros S. (2004). Deformation and failure of the Tymfristos tunnel, Greece. J. Geotech. Geoenviron. Eng..

[10] Peng F. (2010). The application of the laser profiler on the first lining quality inspection of channel. Shanxi Archit..

[11] Novaković G., Lazar A., Kovacic S., Vulic M. (2014). The usability of terrestrial 3D laser scanning technology for tunnel clearance analysis application. Appl. Mech. Mater..

[12] Cheng Y., Qiu W., Lei J. (2016). Automatic extraction of tunnel lining cross sections from terrestrial laser scanning point clouds. Sensors.

[13] Mukupa W., Roberts G.W., Hancock C.M., Al-Manasir K. (2017). A review of the use of terrestrial laser scanning application for change detection and deformation monitoring of structures. Surv. Rev..

[14] Monserrat O., Crosetto M. (2008). Deformation measurement using terrestrial laser scanning data and least squsres 3D surface matching. ISPRS J. Photogramm. Remote Sens..

[15] Xie X., Lu X. (2017). Development of a 3D modeling algorithm for tunnel deformation monitoring based on terrestrial laser scanning. Undergr. Space.

[16] Han S., Cho S., Kim S., Jung J., Heo J. (2013). Automated and efficient method for extraction of tunnel cross sections using terrestrial laser scanned data. J. Comput. Civ. Eng..

[17] Kang Z., Zhang L., Tuo L., Wang B., Chen J. (2014). Continuous extraction of subway tunnel cross sections based on terrestrial point clouds. Remote Sens..

[18] Argüelles-Fraga R., Ordóñez C., García-Cortés S., Roca-Pardiñas J. (2013). Measurement planning for circular cross-section tunnels using terrestrial laser scanning. Autom. Constr..

[19] Qiu W., Cheng Y.-J. (2016). High-resolution DEM generation of railway tunnel surface using terrestrial laser scanning data for clearance inspection. J. Comput. Civ. Eng..

[20] Liu Y., Shi H., Zhu L., Xu X. Research and design of railway clearance measurement system based on non-contact sensors. Proceedings of the 8th World Congress Intelligent Control and Automation (WCICA).

[21] Zhou Y., Wang S., Mei X., Yin W., Lin C., Hu Q., Mao Q. (2017). Railway tunnel clearance inspection method based on 3D point cloud from mobile laser scanning. Sensors.

[22] Du L., Zhong R., Sun H., Zhu Q., Zhang Z. (2018). Study of the Integration of the CNU-TS-1 mobile tunnel monitoring system. Sensors.

[23] Boavida J., Oliveira A., Santos B. Precise long tunnel survey using the RieglVMX-250 mobile laser scanning system. Proceedings of the 2012 RIEGL International Airborne and Mobile User Conference.

[24] Puente I., Akinci B., González-Jorge H., Díaz-Vilariño L., Arias P. (2016). A semi-automated method for extracting vertical clearance and cross sections in tunnels using mobile LiDAR data. Tunn. Undergr. Space Technol..

[25] Amberg Clearance GRP 5000. https://ambergtechnologies.com/solutions-services/amberg-rail/grp-system-fx/.

[26] Leica SiTrack: One. https://leica-geosystems.com/products/mobile-sensor-platforms/capture-platforms/sitrack_one.

[27] Cui H., Ren X., Mao Q., Hu Q., Wang W. (2019). Shield subway tunnel deformation detection based on mobile laser scanning. Autom. Constr..

[28] Kukko A., Kaartinen H., Hyyppä J., Chen Y. (2012). Multiplatform mobile laser scanning: Usability and performance. Sensors.

[29] Du L., Zhong R., Sun H., Wu Q. Automatic monitoring of tunnel deformation based on high density point clouds data. Proceedings of the 2017 ISPRS Geospatial Week.

[30] Du L., Zhong R., Sun H. (2018). Tunnel cross section extraction and deformation analysis based on mobile laser scanning technology. Surv. Mapp..

[31] Sun H., Liu S., Zhong R., Du L. (2020). Cross-section deformation analysis and visualization of shield tunnel based on mobile tunnel monitoring system. Sensors.

[32] Stent S., Gherardi R., Stenger B., Soga K., Cipolla R. (2013). An image-based system for change detection on tunnel linings. Mach. Vis. Appl..

[33] Zhan D., Yu L., Xiao J., Chen T. (2015). Multi-camera and structured-light vision system (MSVS) for dynamic high-accuracy 3D measurements of railway tunnels. Sensors.

[34] Jenkins M.D., Buggy T., Morison G. An imaging system for visual inspection and structural condition monitoring of railway tunnels. Proceedings of the 2017 IEEE Workshop, Environmental Energy and Structural Monitoring Systems.

[35] Zheng E., Wu J., Lv Z. (2014). A non-iterative method for solving the three-dimensional coordinate conversion parameter. Bull. Surv. Mapp..

[36] Code of China (2003). Standard of Metro Gauges. CJJ 96-2003.

[37] Code of China (2013). Code for Design of Metro. GB 50157-2013.

[38] Hormann K., Agathos A. (2001). The point in polygon problem for arbitrary polygons. Comput. Geom..

[39] Least Squares. https://en.wikipedia.org/wiki/Least_squares.

[40] Walon G., Delaloye D., Diederichs M.S. (2014). Development of an elliptical fitting algorithm to improve change detection capabilities with applications for deformation monitoring in circular tunnels and shafts. Tunn. Undergr. Space Technol..

